# Power-Frequency Magnetic Fields and Childhood Brain Tumors: A Case-Control Study in Japan

**DOI:** 10.2188/jea.JE20081017

**Published:** 2010-01-05

**Authors:** Tomohiro Saito, Hiroshi Nitta, Osami Kubo, Seiichiro Yamamoto, Naohito Yamaguchi, Suminori Akiba, Yasushi Honda, Jun Hagihara, Katsuo Isaka, Toshiyuki Ojima, Yosikazu Nakamura, Tetsuya Mizoue, Satoko Ito, Akira Eboshida, Shin Yamazaki, Shigeru Sokejima, Yoshika Kurokawa, Michinori Kabuto

**Affiliations:** 1 National Research Institute for Child Health and Development, Tokyo, Japan; 2 Shakaihoken Funabashichuo Hospital, Funabashi, Chiba, Japan; 3 National Institute for Environmental Studies, Tsukuba, Ibaraki, Japan; 4 Tokyo Women’s Medical University, Tokyo, Japan; 5 National Cancer Center, Center for Cancer Control and Information Services, Tokyo, Japan; 6 Kagoshima University, Kagoshima, Japan; 7 University of Tsukuba, Tsukuba, Ibaraki, Japan; 8 Miyagi University, Kurokawa-gun, Miyagi, Japan; 9 Tokushima University, Tokushima, Japan; 10 Hamamatsu University School of Medicine, Hamamatsu, Shizuoka, Japan; 11 Jichi Medical School, Shimotsuke, Tochigi, Japan; 12 International Medical Center of Japan, Tokyo, Japan; 13 Hiroshima University, Hiroshima, Japan; 14 Kyoto University, Kyoto, Japan; 15 National Institute of Public Health, Wako, Saitama, Japan

**Keywords:** childhood brain tumor, magnetic fields, risk, case-control study, population-based

## Abstract

**Background:**

The strength of the association between brain tumors in children and residential power-frequency magnetic fields (MF) has varied in previous studies, which may be due in part to possible misclassification of MF exposure. This study aimed to examine this association in Japan by improving measurement techniques, and by extending measurement to a whole week.

**Methods:**

This population-based case-control study encompassed 54% of Japanese children under 15 years of age. After excluding ineligible targeted children, 55 newly diagnosed brain tumor cases and 99 sex-, age-, and residential area-matched controls were included in the analyses. The MF exposures of each set of matching cases and controls were measured in close temporal proximity to control for seasonal variation; the average difference was 12.4 days. The mean interval between diagnosis and MF measurements was 1.1 years. The weekly mean MF level was defined as the exposure. The association was evaluated using conditional logistic regression analysis that controlled for possible confounding factors.

**Results:**

The odds ratios (95% CI) for exposure categories of 0.1 to 0.2, 0.2 to 0.4, and above 0.4 µT, against a reference category of <0.1 µT, were 0.74 (0.17–3.18), 1.58 (0.25–9.83), and 10.9 (1.05–113), respectively, after adjusting for maternal education. This dose-response pattern was stable when other variables were included in the model as possible confounding factors.

**Conclusions:**

A positive association was found between high-level exposure—above 0.4 µT—and the risk of brain tumors. This association could not be explained solely by confounding factors or selection bias.

## INTRODUCTION

The association between residential exposure to magnetic fields (MF) and the risk of brain tumors among children was reported in initial epidemiological studies,^1^^–^^3^ but has remained controversial. A review^4^ of 10 previous studies^1^^–^^3^^,^^5^^–^^11^ suggested that inconsistency in the reported associations could be due to bias, or variations in case definition, exposure metrics and cut-off points, control selection methods, and rates of participation.

The present study was conducted in conjunction with a population-based case-control study on childhood leukemia,^12^ in which various methodological improvements were made, particularly with respect to exposure measurements. This study is the first of its kind in Asia, and the results will be included in pooled analysis to be performed in the near future.

## SUBJECTS AND METHODS

The present study was approved by the Ethics Committee for Human Studies of the National Institute for Environmental Studies, Tsukuba, Japan.

### Subjects

**Figure 1.**
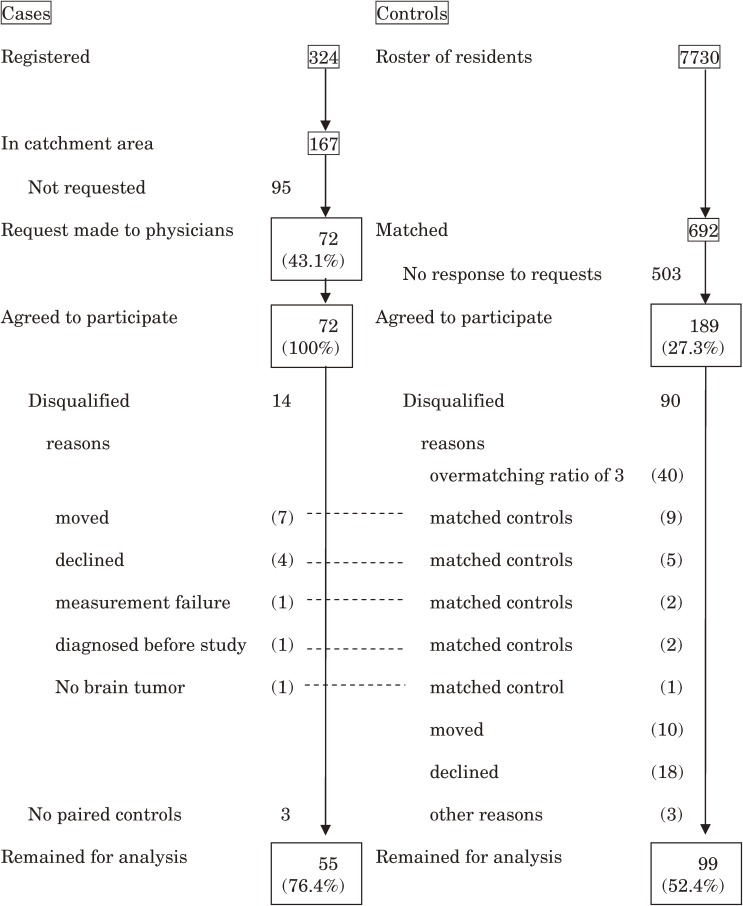
Selection of cases and controls

Patients aged 0 to 14 years with newly diagnosed brain tumors were recruited through a network of 107 hospitals throughout Japan. The diagnosis of brain tumor was histopathologically confirmed by one of the authors (O.K.). The number of cases registered in the study period from May 1999 through September 2002 (2.3 years) was 324, as shown in the flow chart of the selection of cases and controls (Figure 1). Because of limited resources, we set a catchment area for home interview surveys and MF measurements. This catchment area consisted of 5 geographic regions, made up of 18 of the 46 prefectures in Japan: the Kanto area, including Tokyo; the Chukyo area, including Nagoya; the Kansai area, including Osaka; the northern Kyushu area, including Fukuoka and Yamaguchi; and the Hokuriku area, including Toyama and Fukui. The catchment area covered 10.7 million (53.5%) children out of the total population of 20.0 million children aged 0 to 14 years. Among the 324 registered cases, 167 resided in the catchment area. Through their physicians, 72 (43.1%) of these 167 patients were asked to participate, and all agreed to do so. After consultation with their attending physicians, the remaining 95 patients were not asked to participate because the patient had either died before the request or had a serious condition that prevented the attending physicians from asking the families to participate. Of the 72 selected cases, 7 had moved, 4 withdrew after agreement, and measurement failure occurred in 1 case. In addition, we learned at this point that 1 case had been diagnosed before the study period and that, after histopathological confirmation in a central review of the diagnosis, 1 case did not have a brain tumor. Three cases lost their controls because 10 controls moved and 18 withdrew after agreement. After excluding these 17 cases, 55 remained for further analysis.

The control selection procedure is shown in Figure 1. Control candidates were randomly selected from the Japanese resident register in the catchment area. After obtaining informed consent to participate from the patient’s family, we selected controls from a roster of 7730 candidates by matching for sex, age (±25% for age <4 years; ±1 year for age >4 years), region, and the population size of the municipality classified into 4 categories for this study. Because participation rates in mail surveys in Japan do not usually exceed 30%, we selected 10 control candidates for each case to be able to recruit 3 matched controls, which was subsequently changed to 2 matched controls because of limited resources during the study period. A total of 692 matched candidates for the 72 cases in the catchment area were asked to participate. Letters requesting participation were sent as many as 4 times per person until 3 controls (later, 2 controls) were recruited for each case. A total of 189 controls (27.3%) agreed to participate, of which 40 exceeded the matching ratio of 3. Nine controls were excluded from further analysis due to relocation of 7 matched cases, 5 due to withdrawal of 4 cases, and 2 due to measurement failure in 1 matched case. There were 2 matched controls for the 1 case diagnosed before the study period and 1 control for the case that proved not to have a brain tumor; these were also dropped. Ten controls moved and 18 withdrew after agreement to participate, which resulted in the loss of 3 matched cases; 3 controls dropped out for other reasons. After excluding these 90 controls, 99 matched controls remained for analysis.

### Measurements and interview

One-week MF measurements were made in each subject’s bedroom. In addition, 5-minute point-in-time measurements were made in other rooms and in up to 4 corners in the case of detached houses, or both the entrance and opposite side windows in the case of apartment houses. These point-in-time measurements were made to see if the magnetic fields differed at various sites in the house and if the mean of the child’s bedroom levels over the 1-week period was a representative index of the child’s exposure. For the point-in-time measurements, the EMDEX II (Enertech) was used, which measures frequencies from 40 Hz to 800 Hz.

For 1-week measurements, the EMDEX-Lite (Enertech) was placed near the bedside unless the point-in-time magnetic level exceeded 10% of the levels measured at other sites in the bedroom. Accordingly, the instrument was placed away from electric appliances that generated substantial magnetic fields.

The electric currents of power lines yielding MF differ by season; they are highest in summer, followed by winter, mainly due to heavy use of air conditioners and heaters, respectively. Therefore, the date for measuring MF was set near the dates for matched controls in order to control for seasonal variation; the mean difference between cases and controls in a set was 12.4 (standard deviation: 19.9) days.

The weekly arithmetic mean MF level in the child's bedroom was used as the representative exposure index, which was denoted as “bedroom MF level.”

We estimated the distance from each residence to the nearest high voltage power line by using maps provided by 10 power companies in Japan and geographical information system software (ArcInfo, ESRI Inc.). If the distance was less than 100 m, it was measured with a laser-beam distance meter (Yardage Pro Model 20-1000; Bushnell Corp.). This set of information was used to examine possible response bias.

The average time from diagnosis to interview and MF measurement was 1.1 years.

Based on the family’s residential history, we calculated the length of stay in the house where MF levels were measured, and the period from birth to the date of diagnosis, so as to be able to calculate the proportion of time residing in the current house until diagnosis; in controls, the time from birth to the date of diagnosis of the matched cases was used as the length of stay of controls to obtain the proportion of time residing in the current residence.

The questionnaire was a version of that used in the National Cancer Institute study^13^ and was modified for this Japanese study. In brief, the modified questionnaire consisted of demographic profiles, the medical history of family members, history of changes of residence, type of residence, mother’s education, child’s history of vaccinations, mother’s and child’s history of using electric appliances, mother’s history of X-ray examinations during pregnancy, mother’s medication use, smoking, and alcohol drinking, use of pesticides and other chemicals, and mother’s and father’s occupational history. The surveys were mailed to the homes of the study subjects before the home interview.

One or 2 trained interviewers were assigned to each of the 5 blocks in the catchment area. They received training on the purpose and design of the study, the contents of the questionnaire, and the basic techniques of survey interview. The techniques were those of marketing, including the manner of the interview and the proper use of survey cards that show a question and a list of answer choices for the survey items. Training also included role-playing as interviewer and interviewee. The interviewer was instructed to finish the interview in 60 minutes and to spend the same time interviewing cases and controls. The interviewer knew whether the interviewee was a relative of a case or control. Almost all the interviewees were mothers.

### Statistical analyses

The measure of association between the bedroom MF level and childhood brain tumors was the odds ratio (OR) calculated by conditional logistic regression analyses using the PHREG procedures of PC-SAS (Version 8.2; SAS Institute Inc., Japan). The 95% confidence interval (95% CI) of the OR was also calculated.

Cutoff points of 0.1, 0.2, and 0.4 µT, which were used in the pooled analyses by Ahlbom^14^ and in our study on childhood leukemia,^12^ were used in the present study. The use of 0.4 µT as a cutoff point for high exposure in most studies in childhood leukemia was based on the proposal by Ahlbom, who observed an increase of risk around this level in pooled analysis.^14^ Greenland employed 0.3 µT as the cutoff point for high exposure in pooled analysis.^15^ There was therefore no compelling rationale for setting a cutoff of 0.4 µT for childhood leukemia; however, its use has become convention. The evidence for this cutoff is less convincing for childhood brain tumors. However, in a pooled analysis of brain tumors, cutoffs of 0.1, 0.2, 0.3, or 0.4 µT have been employed.^16^ We followed this convention in the present study.

Confounding effects on the risk from bedroom MF were examined by adding selected factors to the logistic regression model as covariates. These included the following: mother’s education: graduate of junior or high school with less than 13 years of education; graduate of college or university with 13 years of education or more. Mother’s history of electric appliance use during pregnancy: electric blanket, heated rug, electric foot warmer, electric sewing machine, hair drier, ultrasonic humidifier, headphones, electric vacuum cleaner, and electric toilet shower (bidet), classified into 4 levels by frequency of use in daily life. Mother’s history of smoking during pregnancy: yes or no. Mother’s history of X-ray examination during pregnancy: yes or no. Child’s history of immunization against poliomyelitis, diphtheria, pertussis, tetanus, measles, rubella, mumps, smallpox, Japanese encephalitis and tuberculosis: yes or no for each. Child’s history of electric appliance use: same as mother’s use of electric appliances, excluding electric sewing machine use.

The mother’s education level was adjusted in all the conditional logistic regression analyses to control for socioeconomic status.

### Evaluation of selection bias

Sensitivity analysis was performed to evaluate the possible influence of selection bias on relative risk parameters due to differential participation. The methodology is briefly described in the Appendix.

## RESULTS

The characteristics of cases and controls are summarized in Table 1. About half the cases were 10 years old or older. The percentages of cases and controls who had lived for 1 year or longer in the house where MF levels were measured were 92.7% and 88.9%, respectively. Only mother’s education was statistically significant, with a higher proportion in cases than in controls of graduates of junior or high schools receiving education of less than 13 years.

**Table 1.** Selected descriptive characteristics for cases and controls

	Childhood brain tumors			
	
	Cases		Controls	
Total number^a^	*n*	%	*n*	%
	55	100.0	99	100.0

Sex				
male	29	52.7	62	62.6
female	26	47.3	37	37.4
Age at diagnosis, y				
≤5	15	27.3	28	28.3
6–9	12	21.8	22	22.2
≥10	28	50.9	49	49.5
Birth order				
1st	7	12.8	18	18.2
2nd	32	58.2	45	45.5
3rd +	16	29.0	36	36.3
Mother’s education^b^				
≤senior high school	26	47.2	30	30.3
≥college/university	28	50.9	60	69.7
Maternal history during pregnancy of:				
1. smoking				
yes	6	10.9	11	11.1
never	49	89.1	88	88.9
2. medical X-ray				
yes	7	12.7	8	8.1
no	41	87.3	91	91.9
3. use of electric appliance^c^				
Residence type				
Single-family house	29	52.7	66	66.7
Apartment house	24	43.6	28	28.3
Child’s use of electric appliance^c^				
Child’s immunization^c^				
“% living”				
<80	27	49.1	42	42.4
≥80	28	50.9	57	57.6
Length of stay at current house before diagnosis, m				
<12	4	7.2	11	11.1
≥12	51	92.7	88	88.9

^a^Totals are not equal to the total number of all cases and controls due to missing data.

^b^Mother’s education level was classified as less than or at least 13 years. Mother’s education level differed significantly (*P* < 0.05) between cases and controls by the chi-square test.

^c^Described in the text.

There was no significant difference between cases and controls in the proportion of those who had received various types of immunization. The proportions of cases and controls were: 90.9% vs 93.9% against poliomyelitis, 92.7% vs 93.9% against DPT (diphtheria, pertussis, and tetanus), 63.0% vs 67.9% against measles, 40.0% vs 39.2% against rubella, 22.2% vs 35.4% against mumps, 25.9% vs 25.0% against MMR (measles, mumps, and rubella), 8.5% vs 12.6% against varicella, 68.9% vs 72.2% against Japanese encephalitis, and 93.5% vs 94.9% against tuberculosis (BCG).

The use of electric appliances among case and control mothers during pregnancy with the study children was as follows. Electric blanket: 9.6% vs 6.1%; heated rug: 45.3% vs 46.5%; electric foot warmer: 11.5% vs 9.1%; electric sewing machine: 21.4% vs 38.4%; hair drier: 81.5% vs 80.8%; ultrasonic humidifier: 3.8% vs 11.1%; headphones: 15.4% vs 15.2%; electric vacuum cleaner: 100% vs 99.0%; toilet shower (bidet): 19.2% vs 25.3%. None of these differences was statistically significant. The proportion of use by case and control children was as follows. Electric blanket: 18.2% vs 6.1%; heated rug: 65.5% vs 52.5%; electric foot warmer: 10.9% vs 15.2%; hair drier: 47.2% vs 51.5%; ultrasonic humidifier: 9.1% vs 11.1%; headphones: 14.6% vs 17.2%. None of these differences was statistically significant.

The correlation coefficients among point-in-time measurements at various locations in the rooms of the residence were mostly higher than 0.85, with no substantial discrepancy between the child’s bedroom and the other rooms in the house as background level. Study children spent the longest time during the day sleeping in their bedroom at home. The mean of 1-week levels in the child’s bedroom was therefore regarded as the best index of the child’s exposure to power-frequency magnetic fields.

The association between bedroom MF level and childhood brain tumors is presented in Table 2. In this analysis, mother’s education, which significantly differed between cases and controls, was controlled in logistic analysis. As compared to the reference category of <0.1 µT, the risk of brain tumor was significantly higher among children whose bedroom MF level was greater than 0.4 µT (OR, 10.9; 95% CI, 1.05–113). The OR for the 0.2 to 0.4 µT category against the same reference was 1.58 (95% CI, 0.25–9.83).

**Table 2.** Association between bedroom MF level and brain tumor

Bedroom MF level (µT)	Childhood brain tumors		

	Cases	Controls	Odds ratio^a^ (95% CI)

	*n* = 55	*n* = 99	
<0.1	47	86	1.0
0.1–0.2	3	8	0.74 (0.17–3.18)
0.2–0.4	2	4	1.58 (0.25–9.83)
≥0.4	3	1	10.9 (1.05–113)

^a^After adjustment for mother’s education level.

The risk associated with a bedroom MF level above 0.3 µT, as opposed to above 0.4 µT, was 16.8 (95% CI, 1.85–153); this is not shown in the table. There were 5 cases and 1 control whose bedroom MF level was above 0.3 µT; 3 cases and no controls in this category resided within 100 m of high voltage power lines (Table 3). Maximum outdoor MF levels were also high in all 5 of these cases. The types of brain tumor were nonglioma (cavernous angioma and mixed germ cell tumor) tumors in 2 cases, and gliomas in 3 cases (astrocytoma in 2 cases and glioblastoma in 1 case).

**Table 3.** Bedroom MF level and characteristics of cases and controls whose bedroom MF level was above 0.3 µT

	Sex	Age at diagnosis (years)	Type of tumor^a^	Bedroom MF level (µT)	Maximum outdoor MF level^b^ (µT)	Residence type	Distance from high voltage power line	Voltage of nearby high voltage power line (kV)
Control	M	6		1.76	0.05	apartment	100+ m	—

Cases	F	11	Nonglioma	2.45	3.94	apartment	<100 m	220
	M	11	Nonglioma	0.51	0.10	apartment	<100 m	275
	M	7	Glioma	0.41	0.26	apartment	100+ m	—
	M	1	Glioma	0.33	0.31	apartment	<100 m	154
	M	0	Glioma	0.34	0.34	apartment	100+ m	—

^a^Type of tumor for the 2 cases of nonglioma was cavernous angioma and mixed germ cell tumor; the tumor type for the 3 cases of glioma was astrocytoma in 2 cases and glioblastoma in 1 case.

^b^Maximum outdoor MF level represents the maximum level of spot measurements at the 4 corners of single-family houses or at the entrance and opposite side windows of apartments.

The risks for proximity to high voltage power lines were slightly higher. As compared to the reference category of greater than 100 m, the OR was 1.88 (95% CI, 0.28–12.8) for residence within 50 m and 1.60 (95% CI, 0.37–6.83) for residence within 50 to 100 m.

When study subjects were restricted to those who had lived longer than 3 months in the current residence, the OR for a bedroom MF level above 0.4 µT became 11.1 (95% CI, 1.06–115); when the duration of residence was longer than 10 months, the OR was 10.1 (95% CI, 0.93–110), with no marked reduction of the risk.

When potential confounding factors were added to the logistic model as covariates, the dose-response pattern was not altered substantially. The odds ratios for high-level exposure (above 0.4 µT) when covariates were added in addition to mother’s education were as follows—child’s history of immunization: 16.9 (95% CI, 1.25–229); mother’s history of X-ray examination during pregnancy: 10.8 (1.05–111); mother’s frequent use of electric appliances during pregnancy (cumulative frequency of the 9 appliances): 13.5 (0.72–253); mother’s smoking during pregnancy: 10.4 (0.88–122); child’s frequent use of electric appliances (cumulative frequency of the 8 appliances): 9.10 (0.69–120). The odds ratios remained relatively stable, although the last 3 odds ratios were statistically nonsignificant.

The results of sensitivity analysis showed that the observed positive association could not be explained merely by possible bias. Even if the maximum possible bias occurred, that is, if all cases within 100 m of power lines had participated, and the control group was free from such bias, the odds ratio was 1.8—well above 1. The details of this analysis are described in the Appendix.

## DISCUSSION

An increased risk for brain tumors was observed among children whose weekly mean bedroom MF level was high. A dose-response pattern in risk was also suggested.

A limitation of this study is its small sample size, which resulted in a very wide confidence interval for the odds ratio in the highest exposure category, ie, above 0.4 µT. Although the odds ratio of 10.9 was statistically significant (the 95% confidence interval did not include unity), a shift of 1 case in the highest exposure category into a lower exposure level would have yielded a nonsignificant OR, markedly below 10.9. Also, when an additional covariate was added, the 3 odds ratios became statistically nonsignificant, although they remained close to the odds ratio that was calculated when only the mother’s education was included in the logistic model. The small sample size in this study also yielded very wide confidence intervals for the odds ratios. This needs to be considered when interpreting and extrapolating the results obtained in this study. In Table 2 we presented the results controlled for mother’s education, an important socioeconomic factor, because only mother’s education was a statistically significant factor in the univariate analysis shown in Table 1, as we have discussed above.

Regarding other limitations, the required study size should have been calculated in advance. For childhood leukemia, the required size was approximately 300 cases and 600 controls if, as assumed, the odds ratio was 2.0 with a power of 0.80 and a significance level of 0.05—values based on the results of the pooled analysis of Ahlbom.^14^ However, there was little information on the expected risk ratio in childhood brain tumors; therefore, the present study did not possess the required study size.

As compared with previous studies,^4^ the advantages of our study include a shorter time between diagnosis and MF measurements, the use of whole-week MF measurements, and the short period between MF measurements within a set of cases and matched controls. These advantages over previous studies may have decreased the effect of some of the limitations discussed above.

The present study focused on residential background power-frequency MF levels in the bedroom. The contribution of electric appliances to the measured level may be of concern. It was reported,^17^ however, that only 3% of the MF level in a high-field home near a transmission line was due to appliance use within the home. Also, the MF level from electric appliances decreased sharply with distance and was below 0.4 µT when the appliances were greater than 1 m away from a resident.^18^ The high bedroom MF levels in the present study, therefore, can be ascribed entirely to MF generated from outdoor sources, such as high voltage power lines or distribution power lines.^19^ A high voltage power line was defined as overhead transmission lines of 22 kV to 500 kV, and a distribution power line was defined as 3-phase 6.6 kV or 7.7 kV overhead distribution wiring.

The arithmetic mean MF level was used, which was the convention in previous studies, although the distribution of residential background fields is usually approximately log-normal and is therefore best summarized by the geometric mean.^17^ In the present study, analyses based on the geometric mean MF level were also performed. As in our previous study on childhood leumekia,^12^ there was no substantial difference between results based on the geometric mean MF and on the arithmetic mean MF.

For most cases and controls, the length of stay in houses where measurements were performed was longer than 12 months. Because the mechanism of tumorigenesis caused by magnetic fields, if such a mechanism exists, is not known, no comment can be made regarding the appropriate length of stay in this type of study. Magnetic fields may act as initiators or promoters; the latter seems more likely if there is a causal relationship. If they act as an initiator, the timing, ie, the age of the children, should be more important than the length of exposure. If MFs act as a promoter, the length of exposure, ie, the length of residence in the home, as was measured in this study, may be related to the magnitude of risk. Brain tumors in this study included gliomas and nongliomas, and their tumorigenesis may differ by histologic type. Another concern is whether a so-called period of susceptibility exists. Many diseases in childhood have a such a period, but it is not certain whether this holds true for the tumorigenesis of brain tumors triggered by magnetic fields; therefore, 12 months, as shown in Table 1, was not chosen as a dividing point related to tumorigenesis but rather as a means to illustrate the proportion of cases and controls with a length of stay over 12 months, which included around 90% of the study subjects.

There should have been very little or no selection bias in the control group. In our study of childhood leukemia,^12^ among the roster of controls, the proportion of those living within 100 m of high voltage power lines was 12.4% in participants and 11.5% in nonparticipants. This lack of selection bias should hold true among the controls in this study on brain tumors, because the roster of controls was the same as that used in the leukemia study. This indicates that the probability of this type of selection bias was very low.

The possibility of selection bias in the case group remains. Of the 72 cases requested to participate, all agreed and hence there was unlikely to have been selection bias at that phase of the study; however, of the 167 cases in the catchment area, participation was requested by attending physicians in only 72 cases (43.1%). The main reasons for declining our request were the serious condition of the patient or a lack of concern among attending physicians. The former reason probably did not introduce selection bias into the risk estimation, but the latter might have. In our opinion, it is not feasible that, when the request to participate was made, the level of exposure to power-frequency magnetic fields was associated with the seriousness of the child’s clinical condition due to brain tumor. No such evidence exists in the literature; however, attending physicians may have become interested in this study if they knew their patients resided close to power lines.

Potential confounding effects were examined but no appreciable modification of the risk of high bedroom MF level was observed; however, the very small numbers of cases and controls in the high exposure category is a limitation, as discussed above.

The present results contrast with those of a recent study in the United Kingdom,^19^ in which no cases and 2 controls had the highest estimated average MF exposure level—above 0.4 µT. In that study, however, the proportion of cases older than 10 years was only 18%, which was much smaller than in the present study (51%) and raises the possibility of different subtype distributions of brain tumors between these studies. As a reference, in the high (above 0.3 µT) bedroom MF level category in the present study, the type of brain tumor found in the 2 cases older than 10 years was nonglioma, while that in the 3 cases aged younger than 10 years was glioma. A comparative study on brain tumor subtypes may be needed to resolve this question.

In addition to the above study, a meta-analysis of 2 studies^10^^,^^20^ in which continuous measurements were taken for 24 hours showed no increased risk of brain tumors for exposure levels above 0.2 µT.^16^ The 3 studies^3^^,^^5^^,^^10^ in which spot measurements were taken showed no increased risk of brain tumors for exposure levels above 0.2 µT in the meta-analysis.^16^ Moreover, the 4 studies^5^^–^^7^^,^^11^ in which calculated fields were used to assess exposure showed no increased risk,^16^ and 2 studies^9^^,^^10^ in which wire was employed to assess exposure showed no increased risk.^16^ In contrast to the above studies, 2 early studies^1^^,^^3^ that used wire to assess exposure showed an increased risk of brain tumors. Reflecting these results, early meta-analyses^21^^,^^22^ indicated an elevated risk of brain tumors, whereas the conclusion of a recent meta-analysis^16^ and review articles^23^^,^^24^ indicated no increased risk.

In summary, an elevated risk of brain tumor was observed in children whose MF exposure level was above 0.3 or 0.4 µT. The association could not be explained solely by selection bias or confounding factors; however, because of the small sample size, the observed increased risk should be interpreted with caution, even though the odds ratio was statistically significant. The results of this study are better appreciated in the context of a recent meta-analysis^16^ than on their own. Detailed pooled analysis is planned and incorporation of raw data from studies performed in Sweden, Norway, Finland, Denmark, Germany, the United Kingdom, the United States, and Japan is underway. In this pooled analysis, not only the association between exposure to magnetic fields and the risk of childhood brain tumors will be analyzed, but also the possibility of bias. This pooled analysis will likely offer a clear conclusion, to which the results of this study will contribute.

**Appendix Table I.** Data layout for sensitivity analysis

Exposure group	Cases	Controls
When all case and control candidates participate		
High	A	B
Low	C	D
(167)		

When participation is limited, as in the observed data		
High	a (3)	b (1)
Low	c (52)	d (98)

Figures in parentheses represent observed numbers.

## APPENDIX

### Sensitivity analysis of selection bias on relative risk parameters

(The methodological details are described elsewhere.^12^)

The data layout of formulations for the evaluation of bias is shown in Appendix Table I, in which 2 situations are assumed: a condition in which all case and control candidates participate and another in which participation is identical to that observed in this study. The true odds ratio estimate, OR_t_, becomes AD/BC from the table and the observed odds ratio estimate, OR_o_, becomes ad/bc. If participation proportions in high and low exposure groups in cases and controls are expressed as PcsH (= a/A), PcsL (= c/C), PcnH (= b/B), and PcnL (= d/D), where cs denotes case, cn denotes controls, H denotes High exposure and L denotes Low exposure, OR_t_ is rewritten as follows.

$${\mathrm{OR}}_{t}=(a/\mathrm{PcsH})(d/\mathrm{PcnL})/(b/\mathrm{PcnH})(c/\mathrm{PcsL})={\mathrm{OR}}_{o}(\mathrm{PcsL}/\mathrm{PcsH})(\mathrm{PcnH}/\mathrm{PcnL})$$

Since it is assumed from the results of the leukemia study, which used the same control population,^12^ that no selection bias exists among controls, ie, PcnH = PcnL, OR_t_ is reduced to (OR_o_)(PcsL/PcsH). Therefore, OR_t_ under selection bias among cases can be evaluated by using hypothetical figures for PcsL and PcsH. When the selection bias is maximal, ie, the true odds ratio is minimal, the numerical Figure 1 is assigned to PcsH. Then, OR_t_ using numerical figures in the tables becomes (3 × 98/52 × 1)(52/164) = 1.8. Here, 164 is the total number requested to participate, 167 minus 3, which is a = A = 3 in the table.

The results of this sensitivity analysis indicate that the true odds ratio is above 1 even when the selection bias among cases was maximal; therefore, the statistically significant odds ratio obtained in this study cannot be explained merely by the existence of possible selection bias.
